# Circular RNA hsa_circ_0043280 inhibits cervical cancer tumor growth and metastasis via miR-203a-3p/PAQR3 axis

**DOI:** 10.1038/s41419-021-04193-7

**Published:** 2021-09-29

**Authors:** Chunyu Zhang, Pan Liu, Jiaming Huang, Yuandong Liao, Chaoyun Pan, Junxiu Liu, Qiqiao Du, Tianyu Liu, Chunliang Shang, Shiyin Ooi, Run Chen, Meng Xia, Hongye Jiang, Manman Xu, Qiaojian Zou, Yijia Zhou, Hua Huang, Yuwen Pan, Li Yuan, Wei Wang, Shuzhong Yao

**Affiliations:** 1 Department of Obstetrics and Gynecology, the First Affiliated Hospital, Sun Yat-sen University, 510080 Guangzhou, Guangdong China; 2 Department of Biochemistry and Molecular Biology, Zhongshan School of Medicine, Sun Yat-sen University, Guangzhou, 510080 China; 3 Department of Obstetrics and Gynecology, Peking University Third Hospital, 100191 Beijing, China

**Keywords:** Prognostic markers, Cervical cancer

## Abstract

Circular RNAs (circRNAs) are known to act as key regulators in a variety of malignancies. However, the role of circRNAs in cervical cancer (CCa) remains largely unknown. Herein, we demonstrated that a circRNA derived from the *TADA2A* gene (hsa_circ_0043280) was significantly downregulated in CCa and that this reduction in expression was correlated with a poor prognosis. Furthermore, our results demonstrated that hsa_circ_0043280 functions as a tumor suppressor to inhibit tumor growth and metastasis in CCa. Mechanistically, hsa_circ_0043280 competitively sponges miR-203a-3p and prevents miR-203a-3p from reducing the levels of PAQR3. Collectively, our results demonstrate that hsa_circ_0043280 plays a pivotal role in the development and metastasis of CCa, thus suggesting that hsa_circ_0043280 has significant potential as a prognostic biomarker and a therapeutic target for CCa.

## Introduction

Cervical cancer (CCa) is the fourth most common female cancer worldwide in 2018, there were ~570,000 new cases of CCa and 311,000 deaths worldwide [1]. Surgery and radiotherapy are the current mainstream strategy for CCa treatment, although the therapeutic effects of these treatments are not ideal for recurrent CCa or the latter stages of this disease [2, 3]. Therefore, it is vital that we gain a better understanding of the precise mechanisms that underlie the progression and metastasis of CCa so that we can develop more specific treatment options.

Circular RNAs (circRNAs) are a new type of regulatory RNA that are characterized by the presence of a covalently closed loop and the lack of a 5′ to 3′ polyadenylated tail [4–6]. Compared to linear RNAs, circRNAs are more stable and resistant to RNase R treatment [7, 8]. CircRNAs have dynamic tissue-specific expression profiles and exhibit different expression levels under different cellular circumstances [9, 10]. Emerging evidence suggests that circRNAs participate in many cellular processes, and increasing evidence now indicates that circRNAs have significant potential to act as competitive endogenous RNAs (ceRNAs) and sponge miRNAs to induce the translation and stability of downstream target genes [11–13]. For instance, circFBXW7 is known to sponge multiple miRNAs and inhibit cell proliferation in many forms of cancers while circCLK3 is known to promote the progression of CCa by sponging miR-320a and enhancing the expression of FoxM1 [14–17]. However, the mechanisms underlying the ability of circRNAs to delicately regulate the progression of CCa remains largely unknown and needs to be investigated further.

In this study, we identified a novel circRNA (hsa_circ_0043280) that acted as a novel tumor suppressor from a previous circRNA microarray data of CCa tissues, and then verified the function of this circRNA with regards to the inhibition of tumor growth and metastasis in CCa. First, we confirmed that hsa_circ_0043280 was downregulated in CCa tissues when compared to normal cervical tissues and then demonstrated that lower expression levels of hsa_circ_0043280 were correlated with a poor prognosis. We also demonstrated that hsa_circ_0043280 could suppress the proliferation, migration, invasion, and metastasis of CCa cells by sponging miR-203a-3p to regulate the expression levels of progestin and adipoQ receptor 3 (PAQR3). Our results indicate that hsa_circ_0043280 exerts anticancer potential and may represent a candidate target for the treatment of CCa.

## Results

### Characteristics of hsa_circ_0043280 in CCa cells

**Fig. 1:**
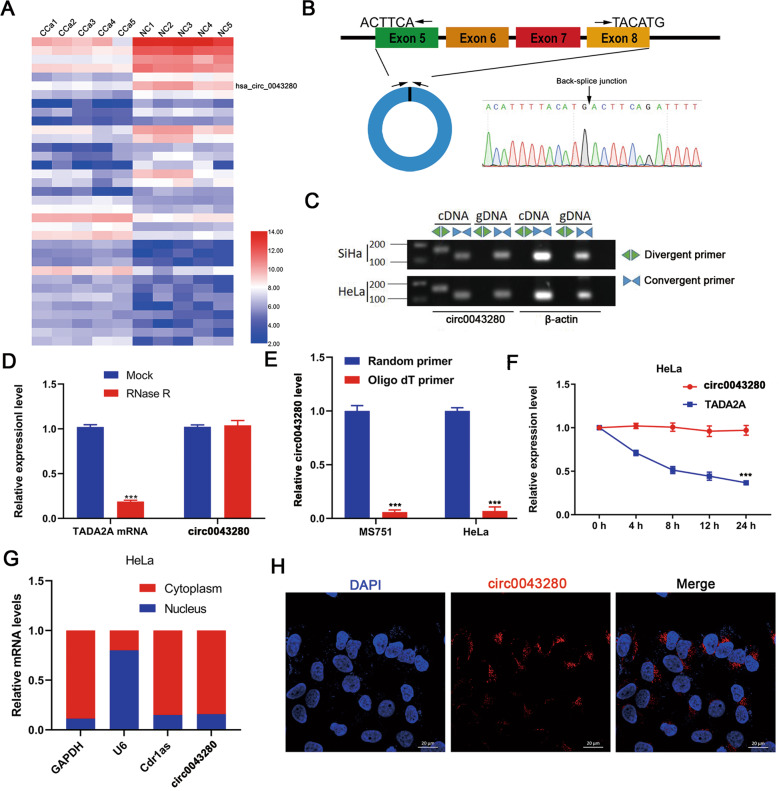
Characterization of hsa_circ_0043280 in CCa cells. **A** The top 35 differentially expressed circRNAs in five pairs of CCa tissues and matched adjacent normal tissues GSE102686, were clustered in a Heatmap matrix. **B** The genomic loci of the hsa_circ_0043280 gene. Hsa_circ_0043280 is synthesized at the TADA2A gene locus containing exons 5 to 8. The back-splice junction of hsa_circ_0043280 was identified by Sanger sequencing. **C** PCR analysis for hsa_circ_0043280 and its linear isoform TADA2A in cDNA and genomic DNA (gDNA). **D** qRT-PCR analysis for the expression of hsa_circ_0043280 and TADA2A mRNA after treatment with RNase R in HeLa cells. **E** qRT-PCR analysis of hsa_circ_0043280 using random primers and oligo dT primers, respectively, in reverse transcription experiments. Hsa_circ_0043280 was notably absent in polyA-enriched samples. **F** qRT-PCR analysis for the expression of hsa_circ_0043280 and TADA2A mRNAs after treatment with actinomycin D at the indicated time points in HeLa cells. **G** Cytoplasmic and nuclear mRNA fractionation experiments showing that hsa_circ_0043280 is localized mainly in the cytoplasm of HeLa cells. GAPDH, Cdr1as, and U6 were applied as positive controls in the cytoplasm and nucleus, respectively. **H** RNA fluorescence in situ hybridization for hsa_circ_0043280 in HeLa cells; the junction probe was complementary to the back-splice junction sequence of hsa_circ_0043280. Nuclei were stained with DAPI. Scale bar, 20 µm. Each experiment was performed at least three times independently. ***P < 0.001.

To gain insight into the role of circRNA in the progression of CCa, we analyzed the expression profiles of circRNAs in five paired samples of CCa along with matched adjacent normal tissues in a previously published circRNA microarray dataset (GSE102686) (Fig. 1A). Since circRNA had a relatively lower abundance in cells than linear mRNA, we chose the top five most abundant differentially expressed circRNAs in cervical tissues; only hsa_circ_0043280, hsa_circ_0031027, and hsa_circ_0065898, were identified in CCa cells (Fig. S1A–C). We also demonstrated that hsa_circ_0043280, hsa_circ_0031027, and hsa_circ_0065898, were downregulated in CCa tissues (Fig. S1D–F). Our preliminary results also showed that the silencing of hsa_circ_0043280, using small interfering RNA (siRNA), had the most significant effect on the proliferation and migration of CCa cells (Fig. S1G–I). Therefore, we chose to focus on hsa_circ_0043280 during this study.

We determined that hsa_circ_0043280 was associated with exons 5 to 8 of the *TADA2A* gene (chr17: 35797838-35804870) and located on chromosome 17q12; hsa_circ_0043280 was 412 nucleotides in length. Next, we amplified the back-spliced junction of hsa_circ_0043280 by designing divergent primers and sequenced this junction by Sanger sequencing (Fig. 1B); this sequence was consistent with that held by the circbase database (http://www.circbase.org/). Next, we used divergent primers for hsa_circ_0043280, and convergent primers for TADA2A, and found that the circular form of hsa_circ_0043280 could only be amplified from cDNA but not from gDNA (Fig. 1C). We were also able to detect hsa_circ_0043280 after RNase R treatment, a protocol that can digest linear RNA (Fig. 1D). Since circRNAs do not possess a 3′ polyadenylated tail, we used oligo dT primers, or random primers, to synthesize reverse transcript products from MS751 and HeLa cells. We then detected the expression levels of hsa_circ_0043280 cDNA and found that hsa_circ_0043280 was undetectable in cDNA derived from oligo dT primers but was successfully detected using cDNA derived from random primers (Fig. 1E). We also found that the expression levels of hsa_circ_0043280 were more stable than TADA2A mRNA following actinomycin D treatment at certain time points (Fig. 1F). To observe the cellular localization of hsa_circ_0043280, we conducted qRT-PCR analysis for nuclear and cytoplasmic hsa_circ_0043280. Results showed that hsa_circ_0043280 was mainly localized in the cytoplasm of HeLa and MS751 cells (Fig. 1G and Fig. S2A); a similar localization pattern was subsequently confirmed in CCa cells by FISH (Fig. 1H and Fig. S2B). Collectively, these findings confirmed that hsa_circ_0043280 exhibited the characteristics of a circRNA and was mainly localized to the cytoplasm.

### The downregulated expression of hsa_circ_0043280 was associated with a poor prognosis in patients with CCa

**Fig. 2:**
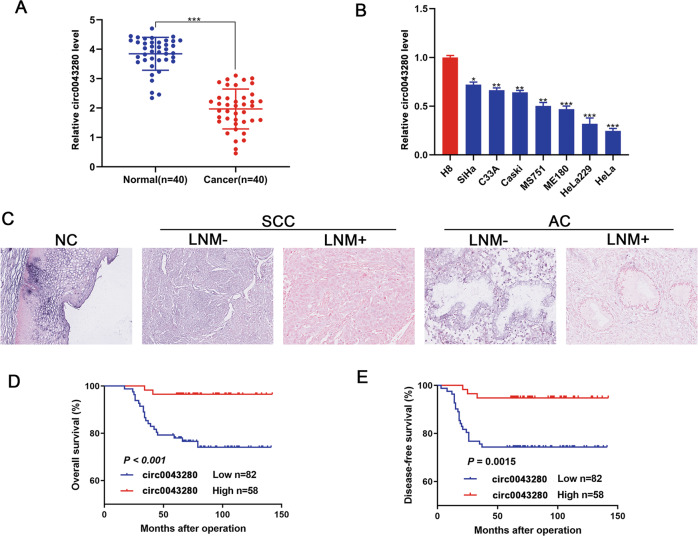
The downregulated expression of hsa_circ_0043280 was associated with a poor prognosis in patients with CCa. **A** Expression levels of hsa_circ_0043280 in CCa tissues in comparison with matched normal tissues were measured using qRT-PCR. **B** qRT-PCR analysis of hsa_circ_0043280 expression in CCa cell lines and a normal cervical cell line H8. **C** ISH analysis of hsa_circ_0043280 in specimens of normal uterine cervical tissues, cervical cancers with lymph node metastasis, and cervical cancers without lymph node metastasis. Original magnification: × 100. Kaplan–Meier survival curves showed poor overall survival (**D**) and disease-free survival (**E**) with low expression levels of hsa_circ_0043280. Each experiment was performed at least three times independently. *P < 0.05; **P < 0.01; ***P < 0.001.

To further investigate the prognostic significance of hsa_circ_0043280 expression in CCa patients, we quantified the levels of hsa_circ_0043280 in a cohort of 40 CCa and 40 normal cervical tissues. Analysis showed that hsa_circ_0043280 was frequently downregulated in CCa tissues when compared to normal cervical tissues (Fig. 2A). Next, we detected the expression of hsa_circ_0043280 in seven CCa cell lines and the normal CCa cell line H8. We found that the expression levels of hsa_circ_0043280 were higher in H8 cells than in the other seven cancer cell lines (Fig. 2B). Next, we further investigated the clinical significance of hsa_circ_0043280 by subjecting 140 CCa samples to in situ hybridization (ISH). Results showed that CCa tissues with lymph node metastasis (LNM) showed lower expression levels of hsa_circ_0043280 than those in CCa tissues without LNM, both for cervical squamous carcinoma and adenocarcinoma (Fig. 2C). Next, we investigated the relationships between hsa_circ_0043280 expression and the clinicopathological characteristics of CCa patients (Table S1). We found in primary CCa samples, lower expression levels of hsa_circ_0043280 were significantly associated with certain prognostic clinical factors, including tumor size (*P* = 0.007) and LNM (*P* = 0.006). Multivariate Cox′s proportional hazards analysis further showed that the expression levels of hsa_circ_0043280, tumor size, FIGO stage, and LNM were independent prognostic factors for CCa (Table S2). Remarkably, Kaplan–Meier survival analysis indicated that patients with lower expression levels of hsa_circ_0043280 were associated with poor overall survival (OS) and disease-free survival (DFS) (Fig. 2D, E). Collectively, our data demonstrated that the expression levels of hsa_circ_0043280 were reduced in CCa, and that this loss of expression was associated with the progression of CCa and a poor prognosis.

### Hsa_circ_0043280 inhibited the proliferation, invasion, and EMT of CCa cells in vitro

**Fig. 3:**
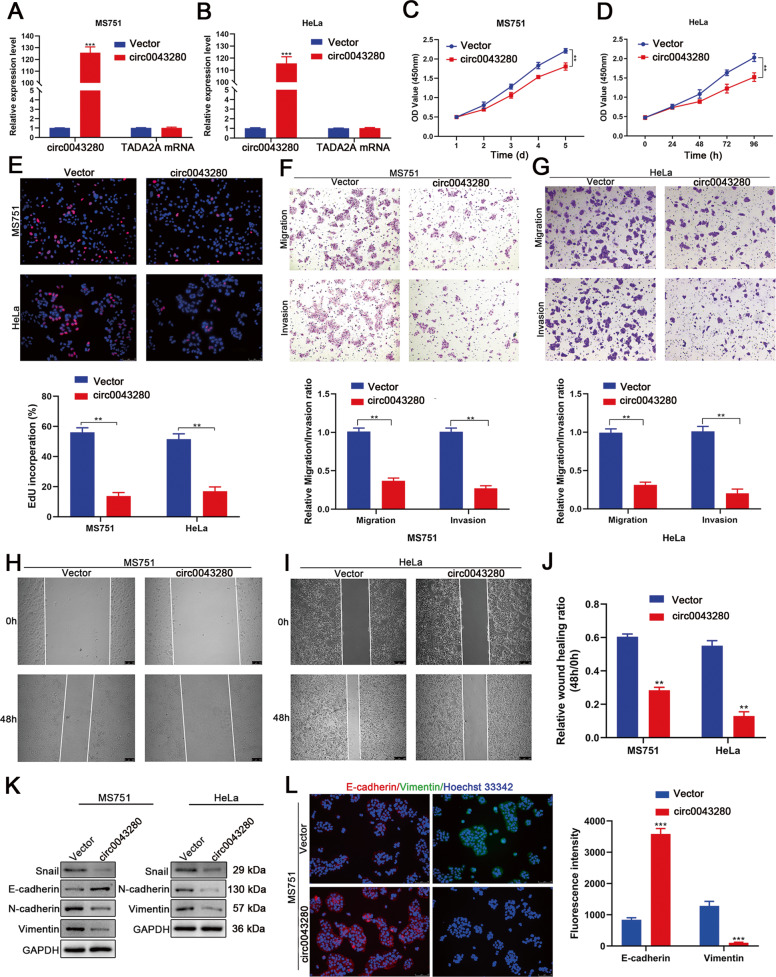
Hsa_circ_0043280 inhibited proliferation, invasion, and EMT in CCa cells in vitro. **A**, **B** Expression levels of hsa_circ_0043280 and TADA2A in MS751 and HeLa cells treated with hsa_circ_0043280 plasmids. The proliferative abilities of MS751 and HeLa cells were measured by the CCK-8 assay (**C**, **D**) and EdU assay (**E**) after the overexpression of hsa_circ_0043280. Original magnification, × 100. **F**, **G** Transwell assays were performed to investigate the effects of hsa_circ_0043280 on the migration and invasion abilities of MS751 and HeLa cell lines. Original magnification, × 100. **H**–**J** Migration abilities were investigated by wound healing analysis of MS751 and HeLa cells transfected with hsa_circ_0043280 overexpression plasmids. Original magnification, × 100. **K** Western blot analysis of EMT markers Snail, E-cadherin, N-cadherin, and Vimentin, in the indicated cells following the overexpression of hsa_circ_0043280. GAPDH was used as a loading control. **L** Immunofluorescence staining verified the upregulated expression of E-cadherin and the downregulated expression of Vimentin in MS751 cells that overexpressed hsa_circ_0043280. Original magnification, × 100. Each experiment was performed at least three times independently. **P < 0.01; ***P < 0.001.

To investigate the effect of hsa_circ_0043280 on the progression of CCa, we considered the expression levels of hsa_circ_0043280 in seven CCa cell lines (Fig. 2B) and selected MS751 cells to perform gain- and loss-of-function assays. We chose these cells because they express moderate amounts of hsa_circ_0043280 and are derived from CCa lymph node metastatic sites. Since SiHa cells retain high basal levels of hsa_circ_0043280 expression, we chose these particular cells for hsa_circ_0043280 loss-of-function assays. HeLa cells were selected for gain-of-function assays because these particular cells had the lowest expression levels of hsa_circ_0043280. The transfection of hsa_circ_0043280 overexpression plasmids, or siRNAs targeting the back-splice region, were shown to efficiently overexpress or knockdown hsa_circ_0043280 without influencing the expression levels of its host gene (TADA2A) in CCa cells (Fig. 3A, B and Fig. S3A), thus indicating that the expression of TADA2A was unaffected by hsa_circ_0043280. Next, CCK-8 and EdU assays demonstrated that the overexpression of hsa_circ_0043280 led to significant suppression of cell proliferation (Fig. 3C–E). Furthermore, Transwell cell migration and invasion assays demonstrated that the ectopic expression of hsa_circ_0043280 led to a dramatic reduction in the motility and invasiveness of CCa cells (Fig. 3F, G). Wound healing assays further revealed that the overexpression of hsa_circ_0043280 inhibited the migration capability of MS751 and HeLa cells (Fig. 3H–J). Conversely, the depletion of hsa_circ_0043280 led to increased viability, migration, and invasiveness, in MS751 and SiHa cells (Fig. S3B-I). It is known that EMT is a crucial process that induces invasiveness in cancer cells. Therefore, we investigated whether hsa_circ_0043280 could repress the EMT phenotype in CCa cells. Western blot analysis showed that MS751 cells that overexpressed hsa_circ_0043280 also expressed higher levels of E-cadherin, an epithelial marker, but lower levels of the mesenchymal markers N-cadherin and Vimentin, and Snail, an EMT transcription factor. Accordingly, the expression of Snail, N-cadherin, and Vimentin, were also reduced in HeLa cells that overexpressed hsa_circ_0043280 (Fig. 3K). In contrast, the silencing of hsa_circ_0043280 led to the downregulated expression of E-cadherin, and the upregulated expression of Snail, Vimentin, and N-cadherin, in MS751 and SiHa cells (Fig. S3J). Immunofluorescence staining further verified the upregulated expression of E-cadherin, and the downregulated expression of Vimentin, in MS751 cells (Fig. 3L), thus indicating that hsa_circ_0043280 could inhibit EMT in CCa cells. Collectively, these results indicated that hsa_circ_0043280 suppresses the proliferation, invasion, and EMT of CCa cells.

### PAQR3 is a downstream target for hsa_circ_0043280

**Fig. 4:**
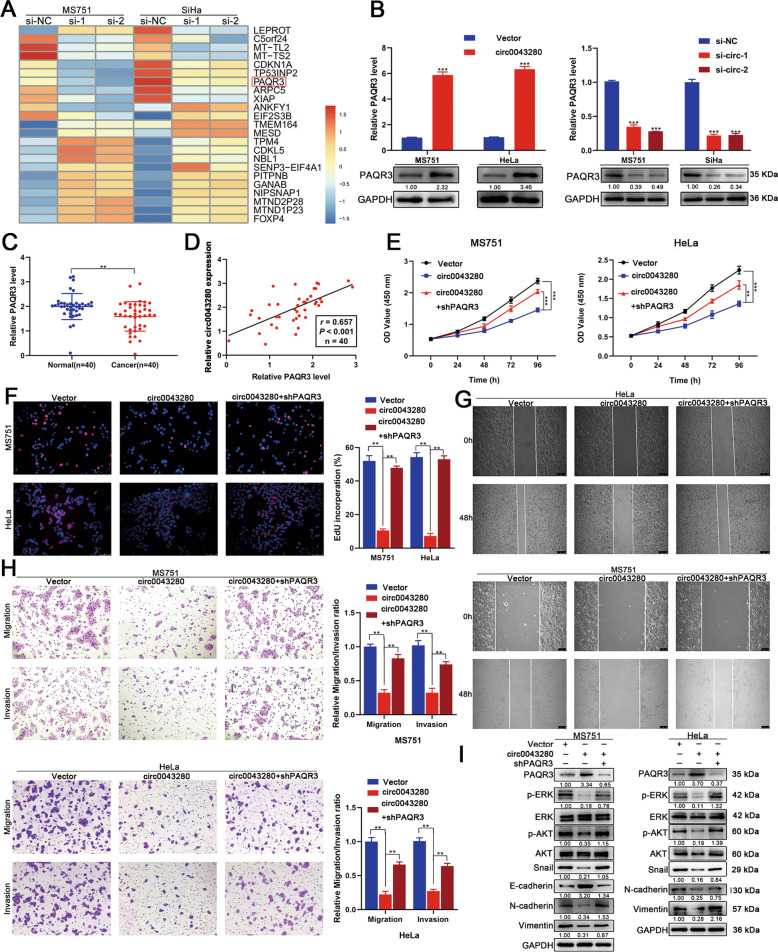
PAQR3 is a downstream target of hsa_circ_0043280. **A** RNA-seq analysis revealed the top 23 differentially expressed genes after the depletion of hsa_circ_0043280 in MS751 and SiHa cells. **B** PAQR3 mRNA and protein expression levels were analyzed by qRT-PCR and western blotting after hsa_circ_0043280 overexpression (left) or ablation (right). **C** Expression levels of PAQR3 in CCa tissues in comparison with normal tissues, as determined by qRT-PCR. **D** Scatter plot analysis of the correlation between mRNA expression levels of hsa_circ_0043280 and PAQR3 in 40 fresh cervical cancer tissues. **E**, **F** Cell proliferation ability was detected by CCK-8 and EdU assays in the indicated cells. Original magnification, × 100. **G** Cell migration abilities were measured by wound healing assays in the indicated cells. Original magnification, × 100. **H** Transwell assays were used to detect the migration and invasion capabilities of the indicated cells. Original magnification, × 100. **I** Western blot analysis of the protein levels of several EMT markers Snail, E-cadherin, N-cadherin and Vimentin, p-ERK, and p-AKT, in the indicated cells. Each experiment was performed at least three times independently. **P < 0.01; ***P < 0.001.

To further investigate the molecular mechanisms that allow hsa_circ_0043280 to suppress the proliferation and aggressiveness of CCa cells, we next performed RNA-seq analysis. Of the differentially expressed genes that were shown to be regulated by hsa_circ_0043280, we observed that CDKN1A, TP53INP2, and PAQR3, were significantly downregulated in MS751 and SiHa cells following the depletion of hsa_circ_0043280 (Fig. 4A). Since CDKN1A, TP53INP2, and PAQR3, were all associated with the development of cancer, we used qRT-PCR to demonstrate that the expression levels of PAQR3 were significantly downregulated by the silencing of hsa_circ_0043280 and dramatically upregulated after the overexpression of hsa_circ_0043280 in CCa cells (Fig. 4B). Next, we validated the downregulated PAQR3 in CCa samples relative to normal cervical tissues using qRT-PCR (Fig. 4C). Although CDKN1A can be regulated by hsa_circ_0043280, this effect was observed to be weaker than for PAQR3. Moreover, there was no significant change in the expression levels of TP53INP2 in MS751 cells that overexpressed hsa_circ_0043280 (Fig. S4A, B). We also detected the correlations between hsa_circ_0043280 and these three genes in a cohort of CCa specimens (*n* = 40). We found that the expression levels of hsa_circ_0043280 were more positively correlated with the mRNA levels of PAQR3 (*r* = 0.657, *P* < 0.001) when compared to those of CDKN1A (*r* = 0.216, *P* = 0.181) or TP53INP2 (*r* = 0.378, *P* = 0.016) (Fig. 4D and Fig. S4C, D). Therefore, PAQR3 was considered as a potential downstream target for hsa_circ_0043280.

PAQR3, a membrane protein of the Golgi body, has been reported to act as a tumor suppressor and negatively regulated to numerous human cancers [18]. Previous literature highlighted that PAQR3 could inhibit cell proliferation, migration, invasion, and EMT, by regulating the PI3K/AKT and Raf/MAPK/ERK signaling pathways in multiple cancers, including prostate, lung, and colorectal cancer [19–23]. We observed that the overexpression of PAQR3 inhibited the proliferation, migration, and invasion, of CCa cells, whereas the depletion of PAQR3 had the opposite effect (Fig. S5A–P). Consistent with previous reports, the overexpression of PAQR3 significantly increased the levels of E-cadherin in MS751 cells but decreased the levels of Snail, N-cadherin, and Vimentin, in MS751 and HeLa cells. Following the knock down of PAQR3 in CCa cells, we observed the opposite effects; these findings highlighted the inhibitory effects of PAQR3 on EMT in CCa cells (Fig. S5Q, R). In addition, we found that PAQR3 could restrain the activation of the AKT and ERK pathways in CCa cells (Fig. S5Q, R).

### Hsa_circ_0043280 suppressed tumor growth and metastasis in CCa by regulating PAQR3

Next, we performed a range of rescue assays to investigate whether hsa_circ_0043280 suppressed the proliferation and aggressiveness of CCa cells in a PAQR3-dependent manner. We found that the knockdown of PAQR3 could abrogate the effects of hsa_circ_0043280 on the suppression of cell proliferation ability, as determined by CCK-8 and EdU assays (Fig. 4E, F). The knockdown of PAQR3 was shown to rescue the inhibition of migration and invasion in MS751 and HeLa cells that overexpressed hsa_circ_0043280 (Fig. 4G, H). Next, we explored whether hsa_circ_0043280 inhibited EMT by modulating PAQR3. As expected, western blot analysis showed that the silencing of PAQR3 dramatically reversed the inhibition of EMT in CCa cells that overexpressed hsa_circ_0043280. Moreover, the inhibition of PAQR3 following the overexpression of has_circ_0043280 led to increased levels of p-AKT and p-ERK expression in MS751 and HeLa cells (Fig. 4I).

**Fig. 5:**
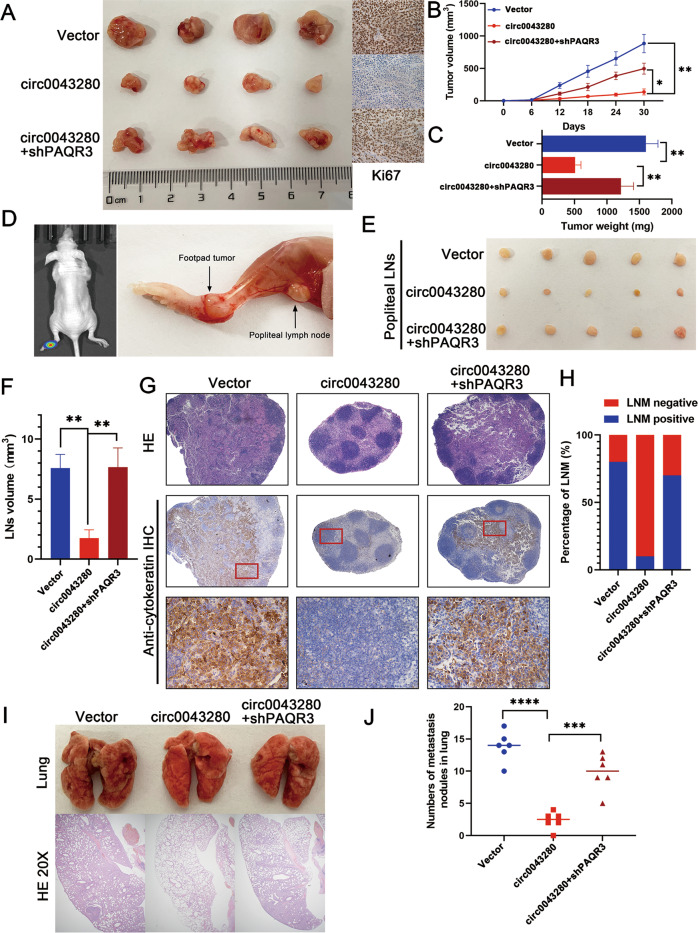
Hsa_circ_0043280 suppresses tumor growth and metastasis in CCa via a mechanism involving PAQR3 in vivo. **A** Representative images of dissected tumors from nude mice transplanted with stable hsa_circ_0043280 overexpression or hsa_circ_0043280 overexpression/PAQR3 knockdown cells. **B** Subcutaneous tumor growth curves of mice in different treatment groups. **C** The mean weight of tumors upon sacrifice in the experimental groups. **D** Representative images of the nude mouse model of popliteal LN metastasis. The indicated CCa cells were injected into the footpads of the nude mice, and the popliteal LNs were enucleated and analyzed. Representative images of enucleated popliteal LNs (**E**) and histogram analysis of the LN volume (**F**) in the indicated cells. **G** Representative images of HE and IHC as indicators of LN status. Original magnification, × 40 for HE and IHC, × 100 (Red). **H** Quantification of popliteal LN metastasis in the indicated groups. Representative images of HE staining of the lungs (**I**) and the number of nodules showing lung metastasis (**J**) in the tail-vein injection nude mice model undergoing different treatments. Original magnification, × 20. Each experiment was performed at least three times independently. *P < 0.05; **P < 0.01; ***P < 0.001; ****P < 0.0001.

The effects of hsa_circ_0043280 on tumor growth and metastasis were further confirmed by animal experiments. A subcutaneous xenograft tumor model indicated that the overexpression of hsa_circ_0043280 led to suppression in tumor growth, while the knockdown of PAQR3 partially reversed the inhibition of tumor growth caused by the overexpression of has_circ_0043280. Furthermore, the depletion of PAQR3 partially enhanced the number of Ki-67 positive cells in subcutaneous tumors when compared with the overexpression of hsa_circ_0043280 (Fig. 5A). Moreover, the knockdown of PAQR3 partially rescued tumor weight and size; these parameters were both suppressed by the overexpression of hsa_circ_0043280 (Fig. 5B, C). We used LNM model to further investigate the role of hsa_circ_0043280 and PAQR3 in LNM in CCa (Fig. 5D). We observed that the volume of the popliteal lymph nodes was significantly smaller in the hsa_circ_0043280 overexpression group than that in the control group and that the PAQR3 ablation group showed a greater nodal volume than that in the hsa_circ_0043280 overexpression group (Fig. 5E, F). The immunostaining of cytokeratin confirmed that overexpression of hsa_circ_0043280 led to a significant inhibition in the metastatic capability of CCa cells to the popliteal lymph nodes and that the loss of PAQR3 rescued this trend, as determined by quantifying the number of metastatic lymph nodes (Fig. 5G, H). In addition, tail-vein assays showed that the overexpression of hsa_circ_0043280 led to a marked reduction in lung colonization by tumor cells, whereas the silencing of PAQR3 led to an increase in the metastatic colonization of the lungs by CCa cells which had been repressed by the overexpression of hsa_circ_0043280 (Fig. 5I, J). Collectively, these results indicated that hsa_circ_0043280 could suppress the proliferation and metastasis of CCa by modulating the expression of PAQR3.

### Hsa_circ_0043280 restored the expression of PAQR3 by sponging miR-203a-3p

**Fig. 6:**
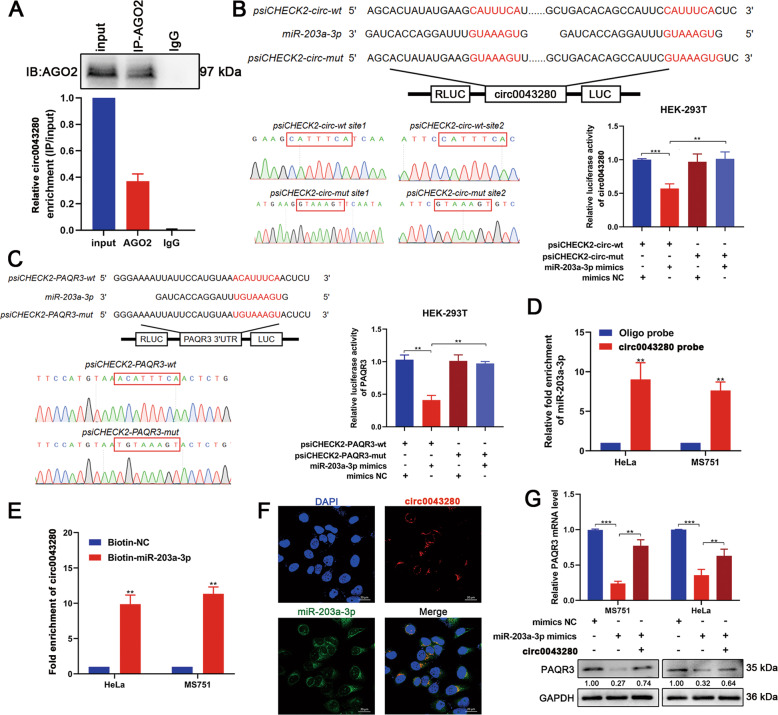
Hsa_circ_0043280 sponged miR-203a-3p to regulate the expression of PAQR3. **A** RIP assay showing the association between AGO2 and hsa_circ_0043280. Top, IP efficiency of the AGO2-antibody in Western blots. Bottom, relative enrichment representing RNA levels associated with AGO2 relative to the input control. An IgG antibody served as a control. **B**, **C** Schematic model and Sanger sequencing for wild-type or mutant transcripts of hsa_circ_0043280 or PAQR3 3′ UTR luciferase reporters (left). Luciferase reporter activity of hsa_circ_0043280 and the PAQR3 3′ UTR in HEK-293T cells co-transfected with miR-203a-3p mimics or mimics NC (right). **D** RAP assays showed that miR-203a-3p could be pulled down by a biotinylated probe designed for the hsa_circ_0043280 back-splicing site in MS751 and HeLa cells. **E** Hsa_circ_0043280 and PAQR3 mRNA were pulled down and enriched with the 3′-end biotinylated miR-203a-3p by miRNA capture assays. **F** RNA FISH indicated the colocalization of hsa_circ_0043280 and miR-203a-3p. Scale bar, 20 μm. **G** PAQR3 expression in MS751 and HeLa cells transfected with miR-203a-3p mimics alone or co-transfected with hsa_circ_0043280. Each experiment was performed at least three times independently. **P < 0.01; ***P < 0.001.

Previous literature showed that circRNAs can sponge miRNAs and subsequently modulate the expression of downstream genes [13, 14]. Since hsa_circ_0043280 was mainly localized in the cytoplasm, we hypothesized that hsa_circ_0043280 might also function as a miRNA sponge to regulate the expression of PAQR3 in CCa cells. To verify this hypothesis, we performed RIP assays using antibodies against AGO2 in cell extracts. We were able to detect hsa_circ_0043280 in AGO2-immunoprecipitated RNAs extracted from MS751 cells (Fig. 6A). Bioinformatic predictions (Circinteractome; Targetscan) indicated that miR-203a-3p had the highest binding affinity with hsa_circ_0043280 and showed conserved binding sites with PAQR3 mRNA. Next, we performed luciferase reporter assays to further confirm that hsa_circ_0043280 and PAQR3 could be targeted by miR-203a-3p. The luciferase activities of hsa_circ_0043280 or PAQR3 wild-type reporters were dramatically reduced when transfected with miR-203a-3p mimics when compared with the mutated luciferase reporter or control group (Fig. 6B, C). Moreover, we were able to pull down miR-203a-3p with the biotinylated probe designed for the hsa_circ_0043280 back-splicing site in both MS751 and HeLa cells, as determined by the RAP assay (Fig. 6D). Next, we used biotin-coupled miR-203a-3p mimics for miRNA capture assays to detect the binding activities of hsa_circ_0043280 and miR-203a-3p. There was an almost ten-fold enrichment of hsa_circ_0043280 in the miR-203a-3p-captured fraction when compared with the negative control (Fig. 6E). Furthermore, RNA FISH revealed that both hsa_circ_0043280 and miR-203a-3p were colocalized in the cytoplasm of MS751 cells, thus indicating that hsa_circ_0043280 could act as a sponge for miR-203a-3p (Fig. 6F). These results suggested that hsa_circ_0043280 can bind to miR-203a-3p, and that PAQR3 mRNA can be targeted by miR-203a-3p. In addition, we also evaluated PAQR3 in CCa cells that overexpressed miR-203a-3p. We found that miR-203a-3p mimics significantly reduced the expression of PAQR3 in MS751 and HeLa cells, although this was rescued by the overexpression of hsa_circ_0043280 (Fig. 6G). Furthermore, miR-203a-3p inhibitors significantly increased the mRNA and protein expression of PAQR3 in CCa cells (Fig. S6A, B), and miR-203a-3p has a higher expression in CCa tissues in comparison with normal tissues (Fig. S6C). We found that the expression levels of miR-203a-3p have a negative correlation with the mRNA levels of PAQR3 (*r* = −0.4459, *P* = 0.004) (Fig. S6E).

**Fig. 7:**
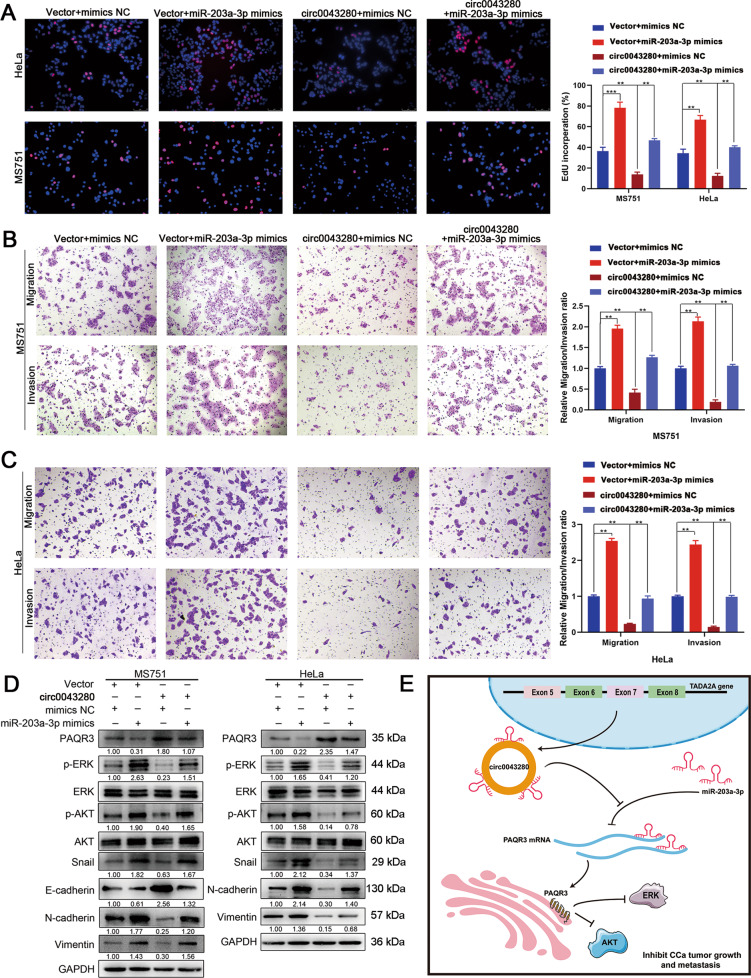
Hsa_circ_0043280 inhibited the proliferation and metastasis of CCa *via* the miR-203a-3p/PAQR3 axis. **A** The proliferative abilities of MS751 and HeLa cells were detected by EdU assays in the indicated cells. Original magnification, × 100. **B**, **C** Transwell assays were performed to investigate the migration and invasion abilities of MS751 and HeLa cell lines in the indicated groups. Original magnification, × 100. **D** Western blot analysis of the protein levels of various EMT markers Snail, E-cadherin, N-cadherin and Vimentin, p-ERK, and p-AKT in the indicated cells. **E** Schematic illustrating the hsa_circ_0043280/miR-203a-3p/PAQR3 signaling pathways in CCa. Hsa_circ_0043280 regulates the expression of PAQR3 by sponging miR-203a-3p and repressing the tumor growth and metastasis of CCa. Each experiment was performed at least three times independently. **P < 0.01; ***P < 0.001.

Next, we performed rescue experiments to further investigate whether hsa_circ_0043280 suppressed cell proliferation and aggressiveness in CCa by interacting with miR-203a-3p. The overexpression of miR-203a-3p enhanced proliferation, migration, invasion, and EMT, in MS751 and HeLa cells, whereas the overexpression of hsa_circ_0043280 rescued miR-203-3p mimics-mediated promotion on cellular phenotype as aforementioned (Fig. 7A–D). We also found that the expression levels of p-AKT and p-ERK could be increased following treatment with miR-203a-3p mimics, and that the overexpression of hsa_circ_0043280 alleviated the activating effects on the ERK and AKT pathways caused by the overexpression of miR-203a-3p in MS751 and HeLa cells (Fig. 7D). Collectively, these data indicated that hsa_circ_0043280 inhibited the progression of CCa by relieving the repression of miR-203a-3p on PAQR3 (Fig. 7E).

## Discussion

In this study, we discovered that hsa_circ_0043280 can regulate CCa via the miR-203a-3p/PAQR3 axis. The development of RNA sequencing and bioinformatics techniques have identified the involvement of a range of circRNAs in multiple malignancies. However, the exploration of the biological functions of circRNAs in the progression of CCa progression is still in its infancy and the mechanisms by which circRNAs influence the development and metastasis of CCa have yet to be elucidated. In the present study, we identified and investigated the crucial inhibitory role of hsa_circ_0043280 in CCa tumor growth and metastasis. We found that the loss of hsa_circ_0043280 expression was associated with a poor prognosis in CCa patients and that hsa_circ_0043280 was an independent predictor for tumor size and LNM in CCa. Moreover, we found that hsa_circ_0043280 could suppress tumor growth and metastasis in CCa by modulating the expression of PAQR3. Mechanistically, we demonstrated that hsa_circ_0043280 restored the expression of PAQR3 by sponging miR-203a-3p. These findings provide new mechanistic insight into the circRNA-mediated inhibition of CCa progression and highlight the potential translational application of hsa_circ_0043280 as an antitumor strategy for the diagnosis and treatment of CCa.

CircRNAs exhibit dynamic tissue-specific expression profiles and functions under different cellular circumstances, including decoy miRNAs or RNA binding proteins (RBPs), some of these can also encode polypeptides [24–30]. But currently, it is most maturely established that circRNAs can effectively sponge miRNAs as they possess high-affinity miRNA-binding sites and a stable circular structure [11, 31, 32]. Previous literature has noted that circRNAs can act as ceRNAs to influence the progression of CCa [17, 33–36]. We found that miR-203a-3p had the strongest binding capability with hsa_circ_0043280 of all alternative miRNAs and that miR-203a-3p possessed a conserved binding site with the 3′UTR of PAQR3 mRNA. We also confirmed that hsa_circ_0043280 could restore the expression of PAQR3 by sequestering miR-203a-3p. Further rescue experiments showed that miR-203a-3p mimics significantly attenuated the repressive effects of hsa_circ_0043280 on the proliferation, migration, invasion, and EMT of CCa cells, thus indicating that hsa_circ_0043280 could suppress tumor growth and metastasis in CCa via the miR-203a-3p/PAQR3 axis.

Accumulating evidence indicates that PAQR3 is downregulated in various tumors and inhibits tumor growth and metastasis by altering pathological signaling pathways [19, 32, 37]. PAQR3 contains seven transmembrane domains and is located on the membrane of Golgi bodies [38]. PAQR3 can sequester Raf kinase to the Golgi apparatus, thereby inhibiting Raf signaling to downstream effectors, thus suppressing Ras/MAPK/ERK and PI3K/AKT signaling pathways [22, 39, 40]. However, whether PAQR3 plays a vital role in the proliferation, mobility, and EMT of CCa cells, has yet to be determined. In the present research, RNA sequencing and western blotting showed that PAQR3 acts as a downstream effector and is regulated by hsa_circ_0043280. We also found that PAQR3 was downregulated in CCa tumors and investigated the potential functional effects of this mechanism in CCa. We found that PAQR3 suppressed cell proliferation, invasiveness, and EMT in CCa. We also found that the overexpression of PAQR3 inhibited the AKT and ERK signaling pathways. Intriguingly, in vivo rescue assays showed that the knockdown of PAQR3 attenuated the inhibitory effects on tumor growth, LNM, and lung metastasis, caused by the overexpression of hsa_circ_0043280. Therefore, our experiments successfully identified the mechanism underlying the tumor suppression caused by hsa_circ_0043280 and expanded our knowledge relating to the function of PAQR3 in the progression of CCa.

## Conclusions

In conclusion, our study revealed that hsa_circ_0043280 was downregulated in CCa cell lines and tissues and that lower expression levels of hsa_circ_0043280 were associated with a poor prognosis. Moreover, hsa_circ_0043280 could competitively bind miR-203a-3p to restore the expression of PAQR3, thus inhibiting tumor growth and metastasis in CCa. Future research involving hsa_circ_0043280 may provide a novel therapeutic approach for the recurrence and metastasis of CCa.

## Materials and methods

### Clinical specimens

CCa tissues and normal cervical tissues were obtained from patients who underwent gynecological surgery between 2009 and 2015 at the First Affiliated Hospital of Sun Yat-sen University (Guangzhou, China). None of the enrolled CCa patients had radiotherapy or chemotherapy prior to surgery and were at stages Ia2 to IIa2 with regular follow-up data; they had also undergone radical hysterectomy and lymphadenectomy. Normal cervical tissues were obtained from patients who underwent hysterectomy under nonmalignant conditions. Upon surgical removal, tissues were immediately frozen in liquid nitrogen and then stored at −80 °C to await RNA extraction. This study was approved by the Ethical Review Committee of the First Affiliated Hospital of Sun Yat-sen University. Patient studies were conducted in accordance with the Declaration of Helsinki.

### Cell culture

Human CCa cell lines (SiHa, HeLa, ME180, C33A, Caski, HeLa229, and MS751) and a normal cervical cell line (H8) were purchased from the American Type Culture Collection (ATCC, USA) and ZQXZBIO (Shanghai Zhong Qiao Xin Zhou Biotechnology Co., Ltd.). Siha, HeLa, and H8 cells were cultured in DMEM (Gibco, China), MS751 and C33A cells were cultured in MEM (Gibco, USA), while HeLa229 and Caski cells were cultured in RPMI1640 (Gibco, USA). All media was supplemented with 10% fetal bovine serum (FBS) (Gibco, USA) and 1% penicillin/streptomycin (Gibco, China). Cells were cultured in a humid atmosphere with 5% CO_2_ at 37 °C. In 2018, all of the cell lines used were tested for authenticity by short tandem repeat (STR) genotyping; the cell lines were also screened for mycoplasma contamination (e-Myco Mycoplasma PCR Detection Kit; iNtRON).

### Xenograft animal models

We purchased female BALB/c nude mice (4–6 weeks of age) from the Experimental Animal Center of Sun Yat-sen University. To create the subcutaneous xenograft tumor model, mice were subcutaneously injected (into the upper back) with 5 × 10^6^ cells diluted in 150 μl of PBS. Tumor growth was then measured every 6 days after injection. Tumor size was measured with calipers and tumor volume was calculated as length × width^2^ × 0.52 [41]. All mice were sacrificed on day 30 after injection. Tumors were then removed, weighed, and embedded in paraffin for IHC staining.

To create the xenograft mouse model of LNM, the cells (1 × 10^6^/50 μl per mouse) were directly injected into the foot pad. On day 28, the mice were euthanized, and the popliteal lymph nodes were enucleated and embedded in paraffin. To create the xenograft mouse model of lung metastasis, the cells (1.5 × 10^6^/150 μl per mouse) were injected intravenously into the tail-vein. At the experimental endpoint, nude mice were anesthetized and sacrificed; their lungs were then resected and fixed with 4% formaldehyde for HE staining. The number of metastatic nodules was counted and diagnosed by specialized pathologists. All experimental procedures were approved by the Institutional Animal Care and Use Committee of Sun Yat-sen University.

### RNA and gDNA extraction, cytoplasmic and nuclear RNA isolation

Total RNAs were extracted from cells or tissues using the SteadyPure Universal RNA Extraction Kit (Accurate Biotechnology (Hunan)Co., Ltd., China) in accordance with the manufacturer’s instructions. gDNA was extracted using the Easypure Genome DNA Kit (Transgen, China). Nuclear and cytoplasmic fractions were isolated using a PARIS Kit (Ambion, Life Technologies, USA). RNA extracted from each of the fractions was analyzed by qRT-PCR to determine the levels of nuclear control transcript (U6), cytoplasmic control transcript (GAPDH, Cdr1as), and hsa_circ_0043280.

### RNase R treatment and actinomycin D treatment

About 5 U/μg of RNase R (Geneseed, China) was incubated with total RNAs for 15 min at 37 °C. The RNA was then purified with an RNeasy Plus Mini Kit (Qiagen, USA). HeLa and MS751 cells were exposed to 2 μg/mL of actinomycin D (Sigma, USA) to block transcription at a given time point. Then, the total RNA was extracted from cells and the stability of hsa_circ_0043280 and TADA2A mRNAs was analyzed by qRT-PCR.

### qRT-PCR, RT-PCR, gel electrophoresis, immunohistochemistry (IHC), western blotting, and hematoxylin-eosin (HE) staining

qRT-PCR, RT-PCR, gel electrophoresis, IHC, western blotting, and HE staining, were performed as described previously [42, 43]. All primers were synthesized by GENEWIZ (Suzhou, China) and primer sequences are given in Supplementary Table S3. GAPDH or U6 were used as internal normalization for qRT-PCR experiments in this study. The primary antibodies used in this study are given in Supplementary Table S4. Immunostained tissue sections were observed under an optical microscope (Leica, DMI6B, Germany).

### Cell transfection and lentivirus transduction

In order to overexpress hsa_circ_0043280 ectopically, we cloned the full length of hsa_circ_0043280 cDNA into the lentiviral pHBLV-CMV-circ-EF1-fLuc-T2A-Puro vector (Hanbio Biotechnology, Shanghai, China). Cells were then infected with the packaged lentivirus and selected with 2 μg/ml of puromycin for 5 days. The pENTER-CMV-His-PAQR3 plasmid was synthesized by Vigene Biosciences (Shandong, China) and were transfected into cells using X-tremeGENE HP DNA Transfection Reagent (Roche, Germany). In order to knock down PAQR3, we purchased lentivirus packaged shPAQR3 from Genepharma (Shanghai, China) and stable transfected cells were screened by puromycin (Sigma, 2 μg/ml) for 5 days. siRNAs and miRNA mimics were purchased from GenePharma and the oligos used are shown in Supplementary Table S3. Transfection was carried out using Lipofectamine RNAiMAX (Invitrogen, USA) in accordance with the manufacturer’s instructions.

### CCK-8 and EdU assays

For the CCK-8 assay, 3 × 10^3^ cells were seeded into 100 μl of complete culture media in 96-well plates for various time periods. A CCK-8 kit (Dojindo Laboratories, Japan) was then used to measure cell viability in accordance with the manufacturer’s instructions. The EdU assay was carried out by using a Cell-Light EdU Apollo567 In Vitro Kit (Ribobio, Guangzhou, China) in accordance with the manufacturer’s instructions. In brief, the cells were incubated with 50 μM of EdU solution for 2 h at room temperature. Following fixation with paraformaldehyde and immersion in glycine solution, the cells were permeabilized with 0.5% Triton X-100 in PBS. Next, the cells were stained at room temperature for 30 min in the dark with Apollo staining solution and Hoechst 33342 reaction solution (Beyotime, Shanghai, China). Finally, cells were washed twice with methanol and PBS. Finally, representative images were acquired by a Leica DMI8 microscope.

### Cell migration, invasion, and wound healing assays

We used a 24-well plate Transwell system to determine cell migration. Chambers (8-μm-pore size, Corning) and Matrigel (BD Science, USA) were used for cell invasion assays and chambers without Matrigel were used for the cell migration assays. About 5 × 10^4^ cells were seeded into the upper chamber and incubated in a 10% FBS complete culture medium for 24 h. Next, the cells were fixed with 4% paraformaldehyde and stained with 0.1% crystal violet. By counting cells under a microscope, it was possible to quantify migration and invasion rates. For the wound healing assay, the cells were seeded into six-well plates and scratched with a 200 μl pipette tip. After 48 h, we used a microscope to acquire representative images; wound areas were then analyzed by Image J. Each experiment was repeated in triplicate.

### Sanger sequencing and next generation sequencing (NGS)

Sanger sequencing of cDNA was performed by Sangon Biotech (Shanghai, China). For mRNA library construction and NGS, we extracted total RNA with Trizol reagent (Invitrogen, CA, USA). The quantity and purity of the total RNA were evaluated with a Bioanalyzer 2100 system and an RNA1000 Nano LabChip Kit (Agilent, CA, USA) with a RIN number >7.0. Poly(A) RNA was purified from total RNA (5 μg) using poly-T oligo-attached magnetic beads and two rounds of purification. Following purification, the mRNA was fragmented into small pieces using divalent cations under an elevated temperature. Then, the cleaved RNA fragments were reverse transcribed to create a final cDNA library using an mRNA-Seq sample preparation kit (Illumina, San Diego, USA) in accordance with the manufacturer’s instructions. The mean insert size for the paired-end libraries was 300 ± 50 bp. Paired-end sequencing was then performed on an Illumina X10 (LC Sciences, USA) in accordance with the manufacturer’s recommended protocol.

### RNA antisense purification assays (RAPs) and miRNA capture assays

A Hsa_circ_0043280 biotinylated probe was designed and synthesized by Synbio Technologies (Suzhou, China). A BersinBio RNA antisense purification assay Kit (BersinBio, Guangzhou) was then used in accordance with the manufacturer’s protocol. In brief, cross-linked cells were lysed, sonicated, and hybridized with the probes at 37 °C for 4 h. Next, the hybridization mixture was incubated with C1 magnetic beads (Invitrogen, USA) at room temperature for 1 h. The bound RNAs were then washed and purified for RNA analysis. For the miRNA capture assay, 3′-end biotinylated miR-203a-3p mimics or control RNA (Genepharma, Suzhou, China) were transfected into CCa cells for 48 h before harvesting. Next, a lysis buffer (150 mM NaCl, 25 mM Tris (pH 7.5), 5 mM DTT, 0.5% IGEPAL, 60 U/mL Superase, 1 × Protease Inhibitor) was added to the cell pellets and incubated on ice for 10 min. The biotin-coupled mRNA–miRNA complex was then pulled down by incubating the cell lysates with streptavidin magnetic beads (Life Technologies) for 1 h at room temperature followed by RNA extraction. The abundance of hsa_circ_0043280 in the bound fraction was then evaluated by qRT-PCR analysis.

### Fluorescence in situ hybridization (FISH) and circRNA in situ hybridization (ISH)

For the FISH assay, a cy3-labeled hsa_circ_0043280 probe and a FITC-labeled miR-203a-3p probe were designed and synthesized by Geneseed (Guangzhou, China). The probe sequences are shown in Supplementary Table S3. Hybridizations were then carried out with a Fluorescent in Situ Hybridization Kit (RiboBio, Guangzhou, China) in accordance with the manufacturer’s protocol. All images were captured by a laser scanning confocal microscope (TCS SP2 AOBS). Formalin-fixed paraffin-embedded tissues were then stained for hsa_circ_0043280 by ISH, as previously described. A biotinylated ISH probe was designed by Synbio Tech (Suzhou, China) for hybridization with hsa_circ_0043280 and signals from the hybridized probes were detected. Staining scores were determined by considering the intensity and proportion of positive cells in five random fields on each tissue section. Scores representing the proportion of positively stained tumor cells in each section were graded as follows: 0, no positive cells; 1, <10% positive cells; 2, 10–50% positive cells; and 3, >50% positive cells. The staining intensity was recorded as 0 (no staining), 1 (weak staining), 2 (moderate staining), and 3 (strong staining). The staining index (SI) was calculated as follows: SI = staining intensity × proportion of positively stained cells; this resulted in scores ranging from 0 to 9. Individual samples were evaluated by two pathologists in a blinded manner, and expression scores of less than or equal to 4 were defined as low expression; samples that were graded as >4 were defined as high expression.

### Dual-luciferase reporter assay

psiCHECK2-circ0043280-mut and psiCHECK2-PAQR3-mut plasmids were designed and synthesized by Hanbio Biotechnology (Shanghai, China). Luciferase activity was measured using a Dual-Luciferase Assay System (Promega) in accordance with the manufacturer’s instructions. Cells were then seeded into 24-well plates at a density of 3 × 10^4^ cells per well. After 24 h, the cells were co-transfected with WT- or MUT-luciferase reporter vectors and miRNA mimics. After 48 h, we measured luciferase activity with a Dual-Luciferase Reporter Assay System. Firefly luciferase activity was used for internal normalization and fold changes were calculated.

### RNA immunoprecipitation (RIP)

RIP experiments were performed as previously described using a Magna RIP RNA-Binding Protein Immunoprecipitation Kit (Millipore, USA). In brief, 2 × 10^7^ CCa cells were cross-linked with 1% formaldehyde and cell extracts were prepared and incubated at 4 °C overnight with an anti-AGO2 antibody. The following morning, RNA–protein complexes were precleared with protein A/G Dynabeads. RNA was then extracted from the complexes with an RNeasy Plus Mini Kit (Qiagen, USA) and analyzed by qRT-PCR.

### Statistical analysis

Statistical analysis was carried out with SPSS version 21.0 and GraphPad Prism version 8.0. Unpaired Student’s *t*-tests were used to analyze differences between two groups. One-way analysis of variance (ANOVA) was used to evaluate differences among multiple groups. The Kaplan–Meier method was used for OS and DFS analysis, and significance was determined by a log-rank test. Multivariate Cox regression analyses were performed to evaluate independent prognostic factors for CCa. The χ^2^ test and Fisher’s exact test were used to analyze the relationships between hsa_circ_0043280 expression and clinicopathological characteristics. Data were presented as the mean ± standard deviation (SD) of at least three independent experiments. A value of *P* < 0.05 was regarded as being statistically significant.

## Supplementary information

Supplementary files

## Data Availability

The datasets generated and/or analyzed during the current study are available from the corresponding author on reasonable request.
