# Supplementary material for: Circular RNA hsa_circ_0043280 inhibits cervical cancer tumor growth and metastasis via miR-203a-3p/PAQR3 axis
Source: Cell Death Dis. 2021 Sep 29;12(10):888. doi: 10.1038/s41419-021-04193-7 (PMC8481253; doi:10.1038/s41419-021-04193-7)
Supplement: Supplementary file 1 — Supplementary files [file 41419_2021_4193_MOESM1_ESM.pdf]

Supplementary Figures and Figure Legends

Fig. S1

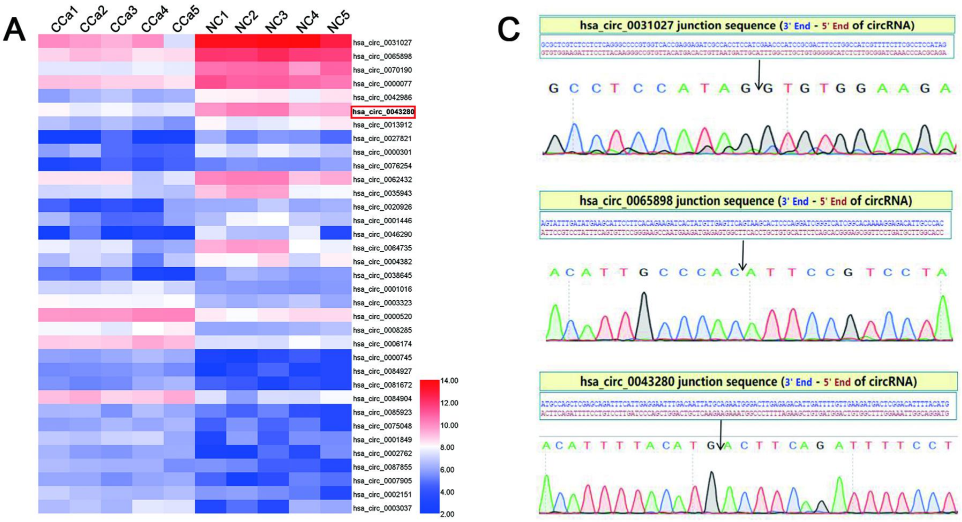

B

circRNA	logFC	Average Expression	t	*P* Value	adj *P* Value	B	gene name
**hsa_circ_0031027**	-4.27880589	11.66951796	-8.553360604	6.22E-06	0.00109721	4.425509766	TMCO3
**hsa_circ_0065898**	-3.14550233	10.63247396	-17.36530973	7.75E-09	2.30E-05	10.33254204	VPRBP
hsa_circ_0070190	-2.92545726	9.31243251	-13.8786229	6.82E-08	0.000100903	8.575780454	BMP2K
**hsa_circ_0043280**	-2.09649356	9.00909721	-8.11293272	9.96E-06	0.001228936	3.965899591	TADA2A
hsa_circ_0000077	-2.03810754	10.0986488	-10.7476702	7.70E-07	0.000691969	6.414603675	TM2D1

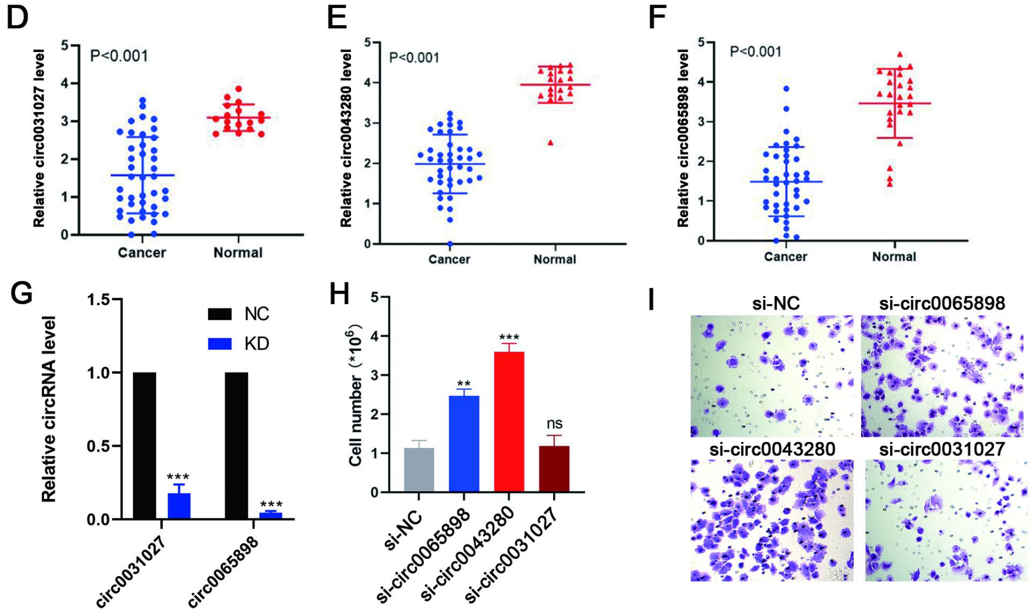

Fig. S1. Screening downregulated circular RNAs in CCa tissues. A The top 35 differentially expressed circRNAs in 5 pairs of CCa tissues and matched adjacent normal tissues were clustered in a Heatmap matrix. **B** The top 5 most abundant circRNAs identified by circRNA microarray in cervix tissues were listed ih this table. **C** The back-splice junction of 3 circRNAs could be identified by Sanger sequencing in CCa cells. **D-F** hsa_circ_0031027, hsa_circ_0043280 and has_circ_0065898 expression were evaluated in CCa and normal cervix tissues. **G-I** The proliferation

and migration abilities of SiHa cells were examined after silencing of circRNAs using small interfering RNA (siRNA). Each experiment was performed at least three times independently. **, $P < 0.01$; ***, $P < 0.001$; ns, no significance.

Fig. S2

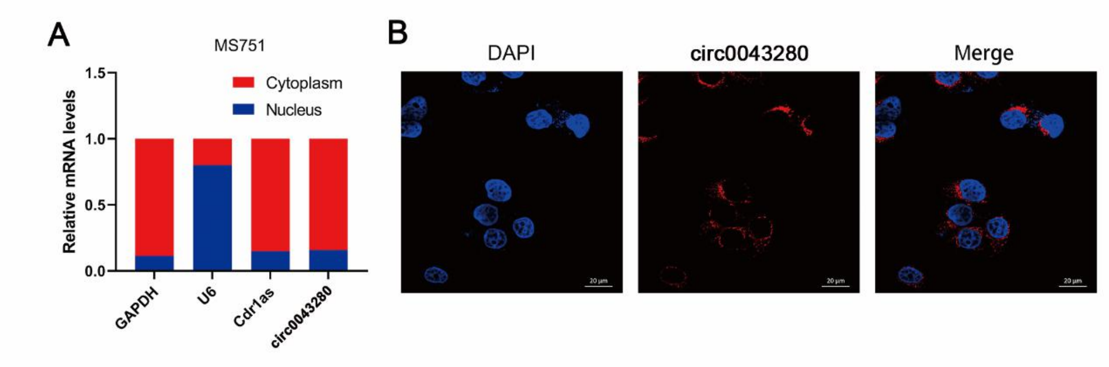

Fig. S2. Hsa_circ_0043280 mainly locates in the cytoplasm of CCa cells. A Cytoplasmic and Nuclear mRNA Fractionation experiment showing that hsa_circ_0043280 localized mainly in the cytoplasm of MS751 cells. GAPDH, Cdr1as and U6 were applied as positive controls in the cytoplasm and nucleus, respectively. **B** RNA fluorescence in situ hybridization for hsa_circ_0043280 in MS751 cells, junction probe is complementary to the back splice junction sequence of hsa_circ_0043280. Nuclei were stained with DAPI. Scale bar, 20 μm. Each experiment was performed at least three times independently.

Fig. S3

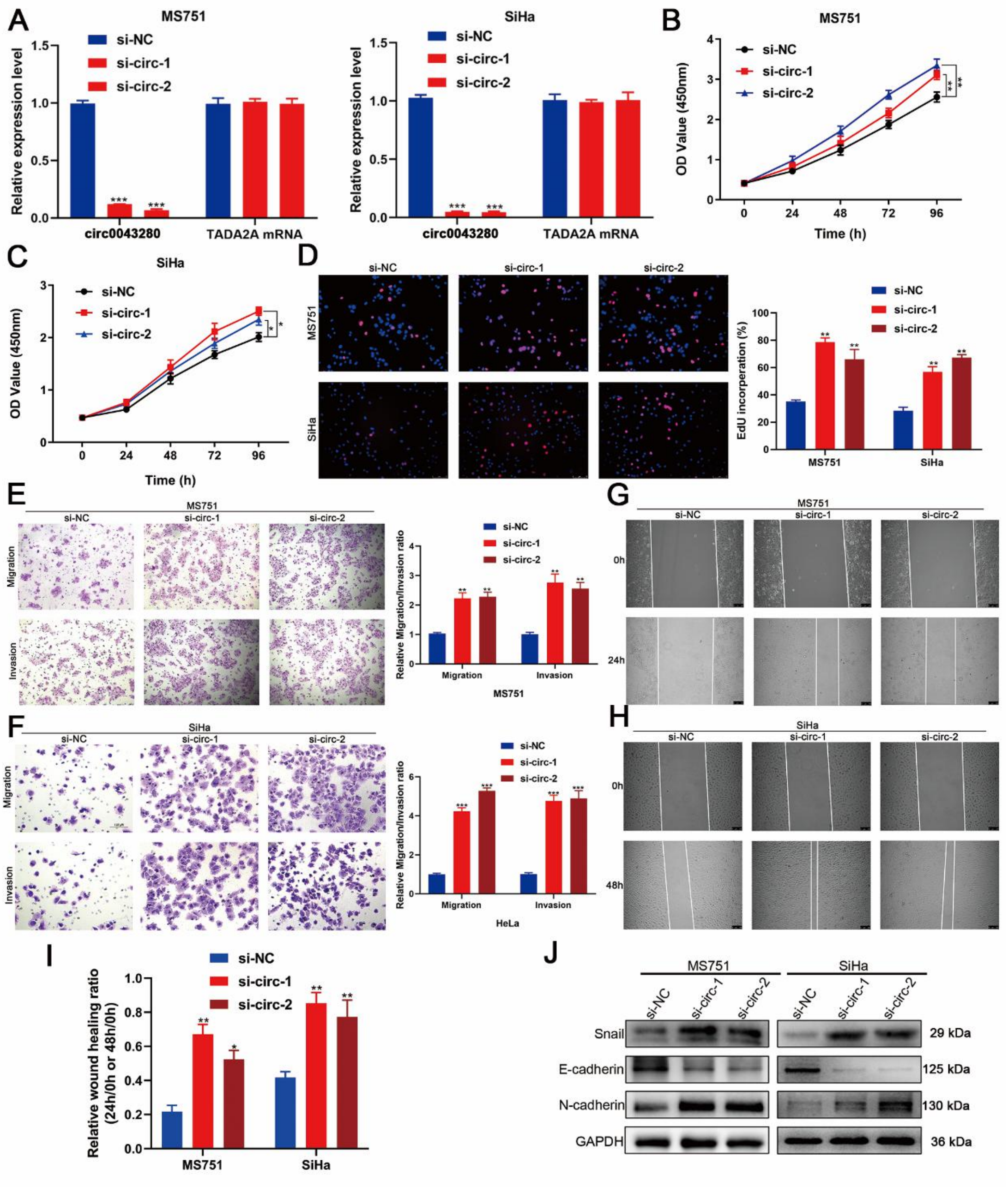

Fig. S3. Knockdown hsa_circ_0043280 promotes CCa cell proliferation, aggressiveness and EMT in vitro. A Expression levels of hsa_circ_0043280 and TADA2A in MS751 and SiHa cells treated with hsa_circ_0043280 siRNAs. The proliferation abilities of MS751 and HeLa cells were measured by CCK-8 assay (**B**, **C**) and EdU assay (**D**) after hsa_circ_0043280 knockdown. Original magnification, × 100. **E**, **F** Transwell assays were performed to investigate the effects of hsa_circ_0043280 knockdown on the migration and invasion abilities of MS751 and SiHa cell lines. Original magnification, × 100. **G-I** The migration abilities were explored by a wound-healing analysis of MS751 and SiHa cells transfected with hsa_circ_0043280 siRNAs. Original magnification, × 100. J, Western blot analysis of

EMT marker (Snail, E-cadherin, N-cadherin and Vimentin) in the indicated cells with ablation of hsa_circ_0043280. GAPDH was used as a loading control. *, $P < 0.05$; **, $P < 0.01$; ***, $P < 0.001$. Each experiment was performed at least three times independently.

Fig. S4

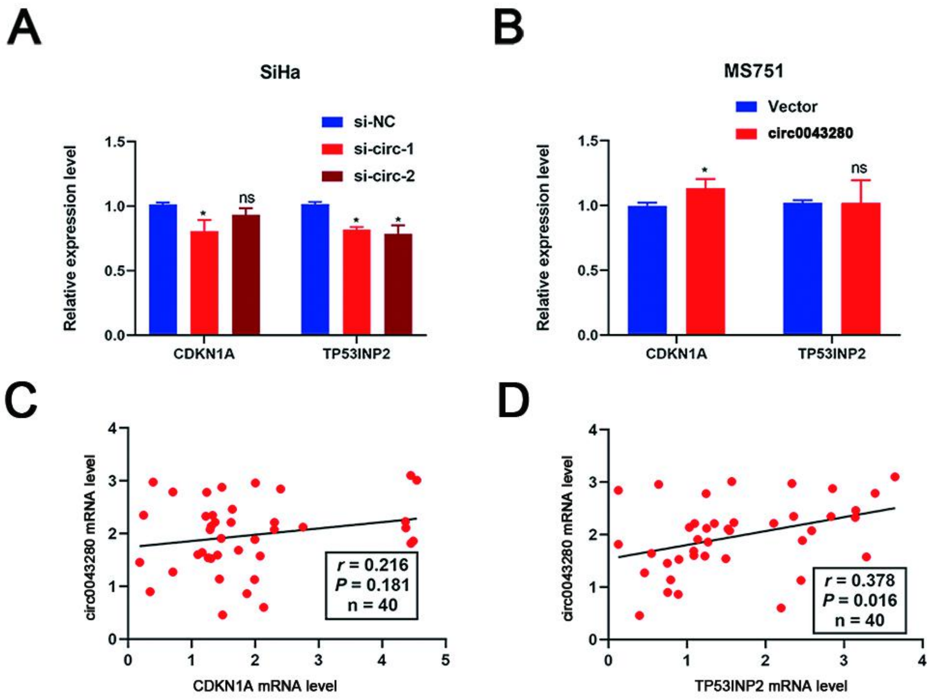

Fig. S4. Detection of mRNA levels of CDKN1A and TP53INP2 in SiHa cells with hsa_circ_0043280 knockdown (**A**) and in MS751 cells with circTP63 overexpression (**B**). The mRNA expression correlation between hsa_circ_0043280 and CDKN1A (**C**) and TP53INP2 (**D**) in specimens cohort (n = 40). * $P < 0.05$; ns, no significance. Each experiment was performed at least three times independently.

Fig. S5

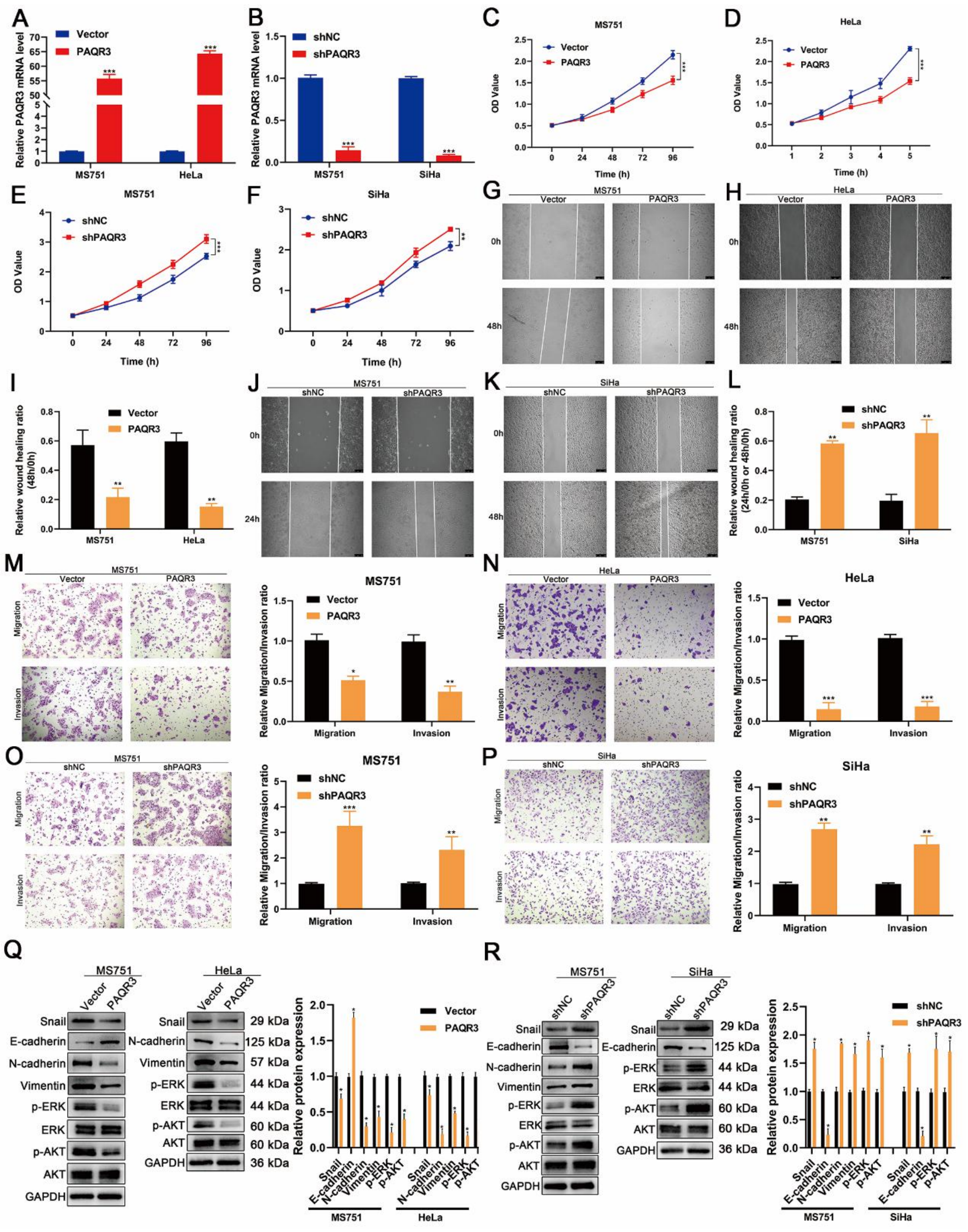

Fig. S5. PAQR3 suppresses CCa cells proliferation, migration, invasion and EMT in vitro. A, **B** qPCR analysis for overexpression and knockdown of PAQR3 in CCa cells. **C-P** gain and loss of function assays were performed to test the proliferation, migration and invasion capabilities of CCa cells manipulated by overexpressing and ablating PAQR3. **Q**, **R** Western bot analysis for the EMT markers (E-cadherin, N-cadherin, Vimentin, Snail), p-AKT and p-ERK expression in the indicated cells. Each experiment was performed at least three times independently. *,$P < 0.05$; **, $P < 0.01$; ***, $P < 0.001$.

Fig. S6

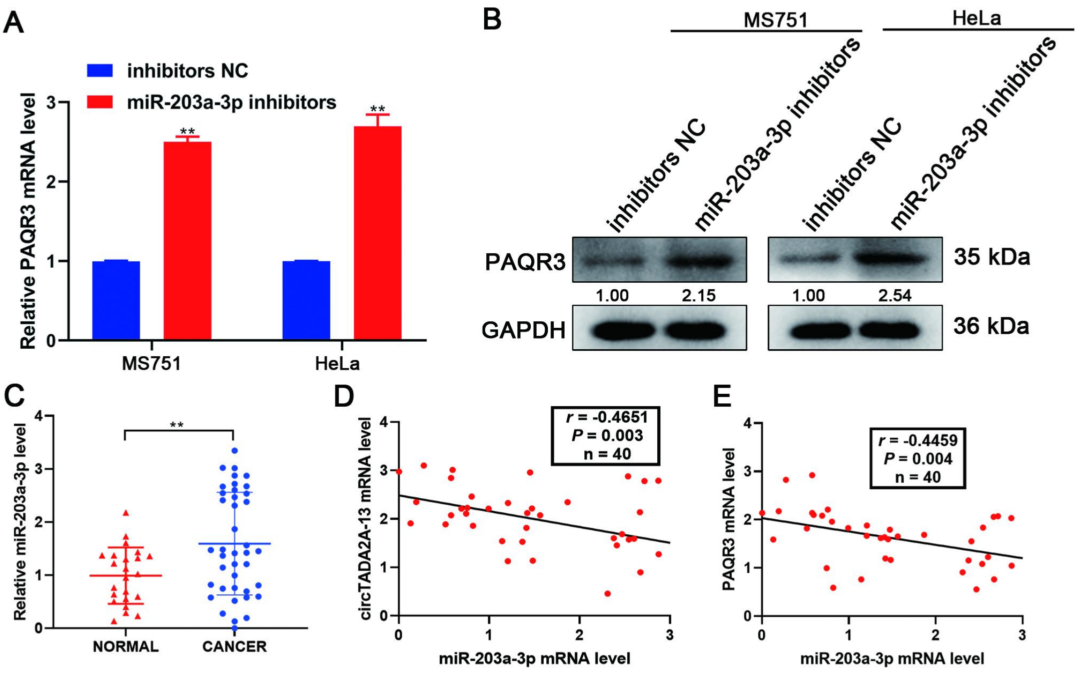

Fig. S6. MiR-203a-3p inhibitors could increase PAQR3 expression levels in CCa cells. A, **B** qRT-PCR and Western bot analysis for the PAQR3 expression in the indicated cells. **C** Expression levels of miR-203a-3p in CCa tissues in comparison with matched normal tissues were measured using qRT-PCR. **D, E** The mRNA expression correlation between miR-203a-3p and hsa_circ_0043280 (**D**) and PAQR3 (**E**) in specimens cohort (n = 40). GAPDH or U6 was used as an negative control. Each experiment was performed at least three times independently. **, $P < 0.01$.

Table S1. Correlation between hsa_circ_0043280 expression and clinicopathologic characteristics of cervical cancer

Characteristics	Total	hsa_circ_0043280 expression		*P*-value
	140	Low	High	
Age (years)				0.458
< 42	60	33	27	
≥ 42	80	49	31	
FIGO stage				0.221
I	114	64	50	
II	26	18	8	
Tumor size (cm)				**0.007***
≤ 4	116	62	54	
> 4	24	20	4	
Pathologic types				0.730
Squamous cell carcinoma	113	68	45	
Adenocarcinoma	21	11	10	
Adenosquamous carcinoma	6	3	3	
Differentiation				0.691
Well	10	6	4	
Moderate	57	31	26	
Poor	73	45	28	
Stromal invasion				0.080
< 1/2	82	43	39	
≥ 1/2	58	39	19	
LVSI				0.052
Positive	19	15	4	
Negative	121	67	54	
LNM				**0.006***
Positive	21	18	3	
Negative	119	64	55	
Vaginal invasion				0.267
Positive	3	3	0	
Negative	137	79	58	
Parametrial invasion				0.511
Positive	2	2	0	
Negative	138	80	58	

χ^2-test. **$P < 0.01$.

FIGO: the International Federation of Gynecology and Obstetrics; LVSI, lymphovascular space invasion; LNM, lymph node metastasis.

Table S2. Multivariate Cox's proportional hazards model analysis of disease-free survival and overall survival

Variables	Overall survival		Disease-free survival	
	HR (95% CI)	*P*-value	HR (95% CI)	*P*-value
Tumor size (cm)		**0.002****		**0.001****
≤ 4 (ref)	1		1	
> 4	4.473 (1.724-11.603)		4.399 (1.825-10.604)	
FIGO stage		**0.028***		**0.024***
I (ref)	1		1	
II	3.041 (1.125-8.222)		2.869 (1.151-7.149)	
LNM		**0.017***		**0.015***
Negative (ref)	1		1	
Positive	2.888 (1.207-6.912)		2.783 (1.218-6.363)	
hsa_circ_0043280		**0.046***		**0.036***
Low (ref)	1		1	
High	0.224 (0.051-0.975)		0.271 (0.080-0.916)	

HR, hazard ratio; 95% CI, 95% confidence interval; ref, reference; For the stepwise multivariate analysis, forward LR method was used to select significant variables. Variables entered for multivariate analysis were the following: Tumor size, FIGO stage, LVSI, LNM, Stromal invasion, hsa_circ_0043280. *$P < 0.05$, **$P < 0.01$.

Table S3. Sequences of primers, siRNA oligos and probes used in the study

primers, siRNA oligos and probes	Sequences
hsa_circ_0043280 (divergent)	Forward 5'-CTACCTTTGACTCCTTGCTT-3'
	Reverse 5'-GACAGGAAAATCTGAAGTCATGT-3'
TADA2A	Forward 5'-CCCCGACCTACCTTTGACTC-3'
	Reverse 5'-TCTCTCAAGTCCCATTCTGCATA-3'
hsa_circ_0043280 (convergent)	Forward 5'-CAAGGAGGAGTGTGAGAAG-3'
	Reverse 5'-AGGTTCAGCAGGGTAGAT-3'
CDR1as	Forward 5'-ACGTCTCCAGTGTGCTGA-3'
	Reverse 5'-CTTGACACAGGTGCCATC-3'
β-actin (convergent)	Forward 5'-TGCCCATCTACGAGGGGTATG-3'
	Reverse 5'-TCTCCTTAATGTCACGCACGATTT-3'
β-actin (divergent)	Forward 5'-AAATCGTGCGTGACATTAAGGAGA-3'
	Reverse 5'-CATACCCCTCGTAGATGGGCA-3'
GAPDH	Forward 5'-ATCACCATCTTCCAGGAGCGA-3'
	Reverse 5'-CCTTCTCCATGGTGGTGAAGAC-3'
PAQR3	Forward 5'-AACCCGTACATCACCGACG-3'
	Reverse 5'-TCTGGACGCACTTGCTGAAG-3'
CDKN1A	Forward 5'-TGTCCGTCAGAACCCATGC-3'
	Reverse 5'-AAAGTCGAAGTTCCATCGCTC-3'
TP53INP2	Forward 5'-CGGCTGGCTCATCATTGAC-3'
	Reverse 5'-CAGCTCTCGTCCATCAAGGA-3'
miR-203a-3p	Forward 5'-GTGAAATGTTTAGGACCACTAG-3'
	The 3’ primer for miR-203a-3p is the mRQ 3’ Primer supplied by Mir-X miRNA First-Strand Synthesis Kit (638315, Takara, Japan)
U6	Forward 5'-ACAGATCTGTCGGTGTGGCAC-3'
	Reverse 5'-GGCCCCGGATTATCCGACATTC-3'
miR-203a-3p mimics	Sense 5'-GUGAAAUGUUUAGGACCACUAG-3'
	Antisense 5'-AGUGGUCCUAAACAUUUCACUU-3'
miR-203a-3p inhibitors	5'-CUAGUGGUCCUAAACAUUUCAC-3'
siNC	Sense 5'-TTCTCCGAACGTGTCACGUTT-3'

	Antisense 5'-ACGUGACACGUUCGGAGAATT-3'
si- circ0043280#1	Sense 5'-CGGACAUUUUACAUGACUUTT-3'
	Antisense 5'-AAGUCAUGUAAAAUGUCCGTT-3'
si- circ0043280#2	Sense 5'-CAUUUUACAUGACUUCAGATT-3'
	Antisense 5'-UCUGAAGUCAUGUAAAAUGTT-3'
si- hsa_circ_0031027	Sense 5'-CUUCGCCUCCAUAGGUGUGTT-3'
	Antisense 5'-CACACCUAUGGAGGCGAAGTT-3'
si- hsa_circ_0065898	Sense 5'-GACAUUGCCCACAUUCCGUTT-3'
	Antisense 5'-ACGGAAUGUGGGCAAUGUCTT-3'
shPAQR3	5'-CCGGCCCGTGTAATTGTGATGTATACTCGAGTATACATCACAATTACACGGGTTTTTG-3'
CY3-labeled hsa_circ_0043280 probe for FISH	5'-GGAAAATCTGAAGTCATGTAAAAT-3'
FITC-labeled miR-203a-3p probe for FISH	5'-TAGTGGTCCTAAACATTTCA-3'
Digoxigenin-labeled hsa_circ_0043280 probe for ISH	5'-GGACAGGAAAATCTGAAGTCATGTAAAATGTCCGAGTC-3'
Biotin-labeled hsa_circ_0043280 probe for RAP	5'-GAAGTCATGTAAAATGTCCG-3'
Biotin-labeled scramble oligo probe for RAP	5'-ATAGACGTCTCGATATAGAG-3'

Table S4. Antibodies used in the study.

Antibody	Catalog#	Working concentration	Manufacturer
PAQR3	PA5-24654	1:1000	Invitrogen
GAPDH	10494-1-AP	1:10000	Proteintech
E-Cadherin	20874-1-AP	1:5000	Proteintech
N-Cadherin	22018-1-AP	1:5000	Proteintech
Vimentin	3879S	1:1000	CST
Snail	3879	1:1000	CST
AKT	4691	1:1000	CST
pAKT	4060	1:2000	CST
Ki-67	23709-1-AP	1:6000	Proteintech
ERK	4695	1:1000	CST
pERK	4370	1:1000	CST
Donkey anti-Rabbit IgG Secondary Antibody, Alexa Fluor 488	A-21206	1:1000	Invitrogen
Donkey anti-Rabbit IgG Secondary Antibody, Alexa Fluor 555	A-31572	1:1000	Invitrogen
